# 临床Ⅰ期高龄非小细胞肺癌的治疗进展

**DOI:** 10.3779/j.issn.1009-3419.2011.12.09

**Published:** 2011-12-20

**Affiliations:** 410008 长沙, 中南大学湘雅医院心胸外科 Department of Cardiothoracic Surgery, Xiangya Hospital, Central South University, Changsha 410008, China

**Keywords:** 肺肿瘤, 高龄, 手术, Lung neoplasms, Aged, Surgery

## Abstract

随着人口的老龄化, 高龄肺癌患者的比例在增大。近十几年来肺叶切除加纵隔淋巴结清扫一直是Ⅰ期非小细胞肺癌(non small cell lung cancer, NSCLC)患者的标准术式。近年研究发现Ⅰ期高龄NSCLC亚肺叶切除术可以取得和肺叶切除术相当的远期疗效而且可以保留更多的正常肺组织, 有关Ⅰ期NSCLC的标准术式再次引起争议。Ⅰ期高龄NSCLC患者是一个特殊的群体, 常因机体功能减退或合并有基础疾病而无法耐受开胸手术, 胸腔镜的问世以及立体定向放射治疗技术的发展使患者有了更多的选择。Ⅰ期高龄NSCLC的治疗在朝着个体化和多样化方向发展。

## Abstract (en)

With the aging of the population, the proportion of elderly lung cancer is increasing.More than ten years, lobectomy with mediastinal lymph node dissection has been the standard surgery for stage Ⅰ non-small cell lung cancer (NSCLC).However, recent studies found that sublobectomy could get the same long-term outcome with the lobectomy for elderly clinical stage Ⅰ NSCLC and more normal lung tissue could be retained.It becomes controversial again about the standard surgery of stage Ⅰ NSCLC at present.Elderly stage Ⅰ NSCLC is a special group, who often can not tolerate thoracotomy because of the poor body function and some comorbidities, but the thoracoscopic surgery and the stereotactic radiotherapy technology (STRT) supply them more options.The treatment of eldly stage Ⅰ NSCLC is developing towards to the individualization and diversification.

据统计, 全世界每年有1, 090万新发恶性肿瘤, 其中肺癌占135万, 每年因肿瘤死亡的670万病例中肺癌占118万。非小细胞肺癌(non-small cell lung cancer, NSCLC)约占肺癌的80%, 其中早期NSCLC患者占30%, 如果不接受手术、放疗、化疗等积极的治疗方式, 其自然病程的5年生存率低于10%^[1]^。随着世界人口的增长及老龄化的加速, 高龄肺癌患者逐渐成为肺癌的主体, 目前在诊断为肺癌的患者中80岁以上肺癌患者的比例在增加^[2]^。

高龄肺癌是指年龄≥70岁的肺癌患者。早在20世纪90年代, 日本学者Fujisawa等^[3]^总结了年龄≥70岁和 < 70岁的肺癌患者之间的术后并发症和远期疗效, 认为术后呼吸系统并发症发生率随着年龄增加明显增高, 且高龄组患者术后生存率明显低于低龄组肺癌患者。高龄肺癌患者是一特殊群体, 一方面年龄较大, 机体的各项功能均较年轻人退化, 另一方面多合并有基础疾病, 最常见的有慢性阻塞性肺病、高血压、冠心病、糖尿病等。这些都使得高龄肺癌患者的治疗比相对低龄组的肺癌患者复杂的多, 术前要求对这一类患者进行严密的评估和准备, 有些患者因为机能的减退而不能耐受手术。目前, 有关Ⅰ期高龄NSCLC的治疗方式主要是手术治疗、放疗、化疗以及综合治疗。

## 1 外科治疗

### 1.1 Ⅰ期NSCLC手术方式

对Ⅰ期NSCLC患者应采取肺叶切除还是亚肺叶切除(包括肺段切除和楔形切除)近十年来一直有争议。20世纪90年代, Ginsberg等^[4]^的前瞻性研究奠定了肺叶切除加纵隔淋巴结清扫作为Ⅰ期NSCLC的标准术式的基础。2005年Nakamura等^[5]^发表的*meta*分析认为Ⅰ期肺癌行肺叶切除和限制性肺切除术后1年、3年、5年生存率无统计学差异。

2008年美国国家综合癌症网络(national comprehensive cancer network, NCCN)在NSCLC治疗指南中提到, 对于心肺功能等身体状况良好的Ⅰ期NSCLC患者应行肺叶切除或一侧肺切除, 如果患者肺功能减退可实行肺段切除或楔形切除, 但能否获得治愈存在争议, 淋巴结清扫应至少包括3组N2淋巴结^[6]^。Chamogeorgakis等^[7]^研究后认为楔形切除对Ⅰ期NSCLC患者并不是一个很好的选择, 楔形切除肿瘤局部复发率高。

### 1.2 Ⅰ期高龄NSCLC的外科治疗

肺癌的外科治疗原则是最大限度切除肿瘤组织和最大限度保留正常肺组织。高龄肺癌患者多伴有肺功能的减退或合并有基础疾病, 最大限度的切除肿瘤组织或根治性手术对高龄肺癌患者来说不一定是最好的选择。

Bilfinger等^[8]^研究认为亚肺叶切除是生理机能减退且不能耐受肺叶切除术的Ⅰ期NSCLC患者的适合选择。2009年Kilic等^[9]^回顾性对比分析了78例行解剖学肺段切除和106例行肺叶切除的 > 75岁Ⅰ期NSCLC患者的围手术期情况及远期疗效, 结果发现两组在术后并发症、死亡率以及远期生存率方面无统计学差异。2010年Okami等^[10]^把764例Ⅰa期NSCLC患者分成年龄≥75岁和 < 75岁两组, 对比分析了两组行肺段切除和肺叶切除术后5年生存率、复发率和术后并发症的情况, 结果发现在 < 75岁的相对低龄组中肺段切除的5年生存率明显优于标准的肺叶切除, 然而在≥75岁的高龄组中肺段切除和肺叶切除其术后5年生存率无统计学差异; 两组中行肺段切除术后复发率均较标准肺叶切除高, 术后并发症在两种术式中没有明显的差异。结论认为, 对Ⅰa期高龄肺癌患者来说, 肺段切除被认为是一个合适的选择, 其远期疗效与肺叶切除相比无明显差异。以上研究结果均未显示Ⅰ期高龄NSCLC患者肺段切除术后并发症及生存率方面劣于肺叶切除术。但由于上述研究均是回顾性分析, 且肺癌TNM分期是以前的分期标准, 2009年第七版肺癌TNM分期在很多方面做了相应的调整, Ⅰ期NSCLC肿瘤大小受到了限制(< 5 cm), 而肿瘤大小又是预后因素, 上述研究结论尚需大样本前瞻性研究进一步证实。对于Ⅰ期NSCLC肺叶切除和肺段切除的比较以及Ⅰ期高龄NSCLC患者标准术式的探讨, 目前国际上美国和日本均在进行前瞻性多中心的大样本临床随机对照研究, 尚未有结果报道。

肺癌患者术中淋巴结清扫很重要。目前对Ⅰ期高龄NSCLC患者术式的选择及纵隔淋巴结清扫范围尚无统一标准, 临床上最常用的是系统淋巴结清扫和淋巴结采样两种方式。潘等^[11]^对临床Ⅰa期NSCLC患者淋巴结转移规律及手术淋巴结清扫范围进行了研究并提出了区域选择性淋巴结清扫的假设, 认为临床Ⅰa期NSCLC患者可行区域选择性淋巴结清扫, 即上叶肿瘤在无肺门或隆突下淋巴结转移时可只清扫上纵隔淋巴结而无须清扫下纵隔淋巴结, 中下叶肿瘤无论有无肺门或隆突下淋巴结转移均需要行上、下纵隔淋巴结清扫。Okada等^[12]^研究认为临床Ⅰa期NSCLC患者区域选择性纵隔淋巴结清扫可以取得与系统淋巴结清扫相同的根治效果, 而且前者的创伤更小。

自20世纪90年代电视胸腔镜应用以来, 胸腔镜在肺癌手术中彰显了其独特的优势。20世纪末Kaga等^[13]^研究认为胸腔镜手术是安全可靠的, 并提出对高龄肺癌患者应考虑使用胸腔镜手术。胸腔镜手术对患者最大的好处在于手术创伤小、术后恢复快。高龄肺癌患者常伴有机体功能减退, 开胸手术的应激和创伤使得高龄患者术后恢复较慢。胸腔镜的微创优势也正好适合高龄肺癌患者这一特殊群体。Mikami等^[14]^研究认为胸腔镜手术可以改善高龄肺癌患者心脏的右室功能, 提高右室射血分数。目前胸腔镜下肺癌根治多用于治疗临床Ⅰ期和Ⅱa期的肺癌。Ohtsuka等^[15]^研究认为临床Ⅰ期NSCLC患者胸腔镜下肺叶切除预后良好且围手术期死亡率低(临床分期为Ⅰ期和病理分期为Ⅰ期的NSCLC患者3年无病生存率分别为79%和89%, 围手术期死亡率不到1%)。Oda等^[16]^的研究支持对Ⅰa期NSCLC可以行胸腔镜下限制性肺切除术, 术后病死率低, 预后良好。Yamashita等^[17]^回顾性分析了早期NSCLC患者在完全胸腔镜下肺段切除和肺叶切除术后肿瘤复发情况以及总体生存率情况, 发现两组无统计学差异。由以上研究可以看出, 胸腔镜手术是Ⅰ期高龄NSCLC患者的合适选择, 尤其是对Ⅰa期的高龄肺癌患者, 胸腔镜不仅减少创伤, 还可以行限制性肺切除保留更多的肺组织, 从而提高患者术后肺功能。

综上, 对于可切除的Ⅰ期高龄NSCLC患者的标准术式目前仍有争议, 现在仍以肺叶切除+系统淋巴结清扫或淋巴结采样作为Ⅰ期NSCLC的标准术式, 而亚肺叶切除适用于部分特殊群体。胸腔镜现已广泛应用, 其疗效已得到认可。随着影像技术的发展及外科水平的提高, 越来越多的早期肺癌被发现, 胸腔镜下亚肺叶切除+区域选择性淋巴结清扫可能是未来Ⅰ期高龄NSCLC患者最佳的术式选择。

### 1.3 手术并发症

20世纪90年代, Duque等^[18]^的前瞻性研究结果显示肺癌术后早期手术相关并发症发生率为27.3%, 其中25.1%为心肺并发症, 引起死亡最常见的并发症为呼吸衰竭, 认为肺癌术后并发症主要发生于术前合并有周围血管疾病和胰岛素依赖性的糖尿病患者。

高龄肺癌患者因其机能的减退多合并有基础疾病, 术后并发症发生率也相对较高。高龄肺癌患者术后并发症主要是心肺并发症, 最常见的有心律失常、肺不张、肺部感染等。对于Ⅰ期高龄NSCLC患者来说, 术后并发症情况直接影响患者预后。Groth等^[19]^研究发现Ⅰ期NSCLC患者行肺叶切除后, 随着年龄的增加心血管事件的死亡率相对于肺癌本身的死亡率在增加, 尤其是≥70岁的高龄肺癌患者。

对于高龄肺癌患者来说, 减少术后并发症的发生需要术前严密的评估、心肺功能储备及原发基础病的处理, 术中尽可能保留正常肺组织, 减少创伤, 术后应加强护理。

## 2 放射疗法

对于Ⅰ期高龄NSCLC外科手术是首选的治疗方式, 但部分患者因为心肺功能较差或合并有基础疾病不宜手术, 根治性放射治疗可能是这部分患者最佳的选择。随着立体定向体部放射治疗(stereotactic body radiation therapy, SBRT)等技术的发展及应用, Ⅰ期NSCLC患者行根治性放射治疗后5年生存率也在提高。

SBRT是一种高度个体化的放疗技术, 它联合多种先进的放疗技术在影像指导下给予肿瘤高剂量精确照射, 其实质是低分次、单次大剂量照射。由于SBRT有专门的治疗计划系统, 放射剂量集中在肿瘤靶区, 肿瘤边缘的放射剂量有陡峭的梯度变化, 因此, 相对于常规的外照射法, SBRT治疗减少了对正常肺组织的损伤。

目前, SBRT被认为是不能手术的Ⅰ期NSCLC患者的标准治疗手段, 其疗效及安全性已有相关报道。Timmerman等^[20]^研究发现早期不能手术的高危肺癌患者行SBRT治疗后3年生存率达55.8%。Turzer等^[21]^研究认为SBRT对伴有低体力状态和合并症的早期高龄NSCLC患者是安全的。

射波刀(CyberKnife)立体定向放射治疗是立体定向放射治疗技术的延伸, 与传统的SBRT相比有其独特的优势, 它能在较短时间内采集患者实时的靶区影像, 自动调整直线加速器的位置, 校正误差并跟踪治疗^[22]^。Ahn等^[23]^认为射波刀定向立体放射治疗是一个非常安全且能够达到很高局部控制率的治疗手段, 可以作为早期NSCLC治疗的适合选择。

随着放疗技术的发展和更新, 对Ⅰ期NSCLC患者来说SBRT可以取得和手术治疗相当的远期疗效。Crabtree等^[24]^对Ⅰ期NSCLC患者行外科治疗和SBRT治疗的预后进行了研究, 认为采用两种治疗方式的Ⅰ期肺癌患者, 其肿瘤局部复发率和生存率无统计学差异。Palma等^[25]^对三个不同时期应用SBRT的≥75岁Ⅰ期NSCLC患者进行了研究, 结果发现接受SBRT的患者比例在增加, 其中位生存率从16个月增加到了21个月, SBRT治疗后的总体生存率和手术治疗相比无明显差异。肿瘤实时跟踪SBRT的应用使得早期高龄NSCLC患者在1年-2年内肿瘤局部控制率高达96%, 且副作用很小, 患者治疗后生活质量高^[26]^。

综上, 对Ⅰ期高龄NSCLC中无法行手术治疗或不愿接受外科治疗的Ⅰ期的高龄NSCLC患者来说, 肿瘤实时跟踪立体定向放射治疗及CyberKnife立体定向放射治疗是一个理想的选择。

## 3 化学疗法

手术治疗NSCLC有一定的复发率, 辅助化疗在术后显得尤为重要。辅助化疗可提高术后5年生存率的优势使其被Ⅱ期和Ⅲa期的肺癌患者广泛接受, 但对Ⅰ期肺癌患者来说辅助化疗的疗效尚存在争议^[27]^。2010年NCCN肺癌治疗指南中, 对Ⅰ期NSCLC不推荐使用化疗。对于Ⅰa期患者术后切缘阳性者化疗作为辅助治疗(2B类), 对于Ⅰb期NSCLC患者化疗仅用于高危患者(2B类)^[6]^。因化疗成本高且副作用相对较大, 化疗仅作为部分Ⅰ期高龄NSCLC患者的辅助治疗。

## 4 综合治疗

临床中由于各种因素, 手术、放疗、化疗的不同组合方式的综合治疗较单纯的治疗更常见。手术+化疗或放疗+化疗组合是最常见的治疗模式。

目前, 对Ⅰa期患者来说不主张术后化疗, 而Ⅰb期患者术后是否行化疗尚存在争议^[28]^。Felip等^[29]^对比研究了术前化疗+手术、手术+辅助性化疗与单独手术治疗对早期NSCLC患者无病生存率的影响, 结果发现各组间无统计学差异。2011年Yamashita等^[30]^研究认为Ki-67(反应肿瘤增殖的一种标记物)标记指数是Ⅰ期NSCLC患者胸腔镜下肺段切除术后无病生存率的预后指标, 对术后Ki-67阳性者应予以辅助化疗。

部分因肺功能差而不能耐受肺叶切除的Ⅰ期高龄患者, 在行限制性肺切除术后辅以放疗的研究临床上也有报道。2010年Blasberg等^[31]^报道了机器人^125^Ⅰ粒子植入放射疗法用于不能行肺叶切除的高危Ⅰ期NSCLC患者, 在行亚肺叶切除术后应用机器人^125^Ⅰ粒子植入放射疗法可减少肿瘤的复发率。

## 5 总结及展望

对肺癌应实行三级预防, 减少其发生的危险因素, 早发现、早治疗, 提高肺癌患者术后生存率及生活质量。对于Ⅰ期高龄NSCLC患者的治疗, 需综合分析患者的病情(包括肿瘤分期、定性、定位等)和患者自身情况(包括患者自身的基础病、机体功能等), 以便于制定适合患者的个体化综合治疗方案。

目前, 手术治疗仍是Ⅰ期高龄NSCLC患者的首选治疗。对高龄肺癌患者来说最大限度的保留正常肺组织尤为重要, 限制性的肺切除及胸腔镜微创治疗是今后外科治疗的发展方向, 它可以减少手术创伤面积和术后并发症, 提高患者术后生活质量。随着放射治疗技术的发展和成熟, SBRT是机体功能较差且不能耐受手术的Ⅰ期高龄NSCLC患者的标准治疗手段, 认识和接受其治疗的患者比例在增加。分子生物学水平在飞速的发展, 药物分子靶向治疗也取得了很大的进步。受化疗副作用及疗效的限制, 对于Ⅰ期高龄NSCLC患者化疗仅作为辅助治疗用于临床。总之, Ⅰ期高龄NSCLC是肺癌中的特殊群体, 对这部分患者应采用个体化的综合治疗。
